# Upper Esophageal Sphincter Metrics across Eosinophilic Esophagitis, Gastroesophageal Reflux Disease and Functional Dysphagia: A Pilot Study

**DOI:** 10.3390/jcm12175548

**Published:** 2023-08-25

**Authors:** Luigi Ruggiero, Paola Iovino, Domenico Gargano, Angela Caloro, Luca De Leo, Antonio D’Antonio, Alessandro Caputo, Antonella Santonicola

**Affiliations:** 1 Gastrointestinal Unit, Department of Medicine, Surgery and Dentistry “Scuola Medica Salernitana”, University of Salerno, 84084 Baronissi, Italy; dr.luigiruggiero@gmail.com (L.R.); piovino@unisa.it (P.I.); angelacaloro97@gmail.com (A.C.); lucadeleo98@gmail.com (L.D.L.); 2 Allergy and Clinical Immunology Unit, San Giuseppe Moscati Hospital, 83100 Avellino, Italy; dogargano4920@aosgmoscati.av.it; 3 Pathologic Anatomy Unit, Department of Medicine, Surgery and Dentistry “Scuola Medica Salernitana”, University of Salerno, 84084 Baronissi, Italy; ada66@inwind.it (A.D.); alcap94@gmail.com (A.C.)

**Keywords:** esophageal diseases, eosinophilic esophagitis, upper esophageal sphincter, high-resolution manometry, GERD, functional dysphagia

## Abstract

Background: Recent studies have evaluated the upper esophageal sphincter (UES) with high-resolution manometry (HRM) in some esophageal diseases, but not eosinophilic esophagitis (EoE). The aim of our study was to evaluate the function of the UES across EoE, gastroesophageal reflux disease (GERD), functional dysphagia (FD), and the relationship with esophageal symptoms, esophageal body contraction, and esophagogastric junction (EGJ) metrics. Methods: HRM was performed on 30 EoE, 18 GERD, and 29 FD patients according to the Chicago Classification 3.0. The study data were exported to the online analysis platform Swallow Gateway. The UES was assessed in terms of UES Resting Pressure (UES-RP), UES Basal Pressure (UES-BP), UES Integrated Relaxation Pressure (UES-IRP), UES Relaxation Time (UES-RT), Basal UES Contractile Integral (Basal UES-CI), Post-Deglutitive UES Contractile Integral (Post-Deglutitive UES-CI), and Proximal Contractile Integral (PCI). Results: ANOVA analysis showed significantly higher values of Post-Deglutitive UES-CI in EoE patients compared with FD patients (*p* = 0.001). Basal UES-CI and UES-RP showed significantly higher values in EoE (*p* = 0.002, *p* = 0.038) and GERD (*p* < 0.001, *p* = 0.001) patients compared with FD patients. Correlations between LES-CI and Post-Deglutitive UES-CI, Basal UES-CI, and UES-RP (*p* ≤ 0.001, *p* = 0.027, *p* = 0.017, respectively), and between LES-BP and Post-Deglutitive UES-CI (*p* = 0.019), independent of diagnosis, were shown. No correlations have been demonstrated between the UES, EGJ metrics, and esophageal symptoms. Conclusions: Some differences in UES metrics in the three different diseases were found. Further studies are needed to confirm the results of our pilot study and possible applications in clinical practice.

## 1. Introduction

The upper esophageal sphincter (UES) is a high-pressure zone separating the pharyngeal lumen from the esophageal lumen. It consists of parts of the cervical esophagus (CE), the cricopharyngeus (CP), and the inferior pharyngeal constrictor (IPC) [1]. One of the main functions of the UES is to prevent esophago-pharyngeal reflux; however, it also plays a key role during swallowing, belching, and vomiting [2]. UES tone can be modified by a range of physiological and pathological conditions. It decreases with swallowing, belching, vomiting, expiration, and sleep. It is also decreased in infants and elderly people. It increases with inspiration, stress, esophageal distension, intra-esophageal acid, secondary peristalsis originating in the esophagus, and pharyngeal stimulation with air or water [3]. The UES is usually studied by videofluoroscopy, although high-resolution manometry (HRM) is beginning to play an increasingly important role. In fact, some recent studies have investigated the UES using HRM in achalasia (Ach) [4,5] and esophagogastric junction outflow obstruction (EGJOO) [6], demonstrating that some metrics of the UES can help to differentiate among Ach subtypes as well as shed some light on etiologies underlying EGJOO. Another recent study used HRM to investigate the UES function in patients with gastroesophageal reflux disease (GERD), showing a short and hypotonic UES [7].

Eosinophilic esophagitis (EoE) is a chronic immune/antigen-mediated disorder characterized by symptoms of esophageal dysfunction (i.e., dysphagia and bolus impaction) and, histologically, by eosinophilic inflammation in the absence of secondary causes of eosinophilia [8]. Using HRM, several alterations in esophageal motility were found in patients with EoE, such as EGJOO, ineffective esophageal motility (IEM), distal esophageal spasm (DES), hypercontractile esophagus (HE), and Ach, and many of these alterations often resolved after surgical or drug therapy [9,10]. However, to our knowledge, UES metrics in patients with EoE have not been studied using HRM, nor have the causes of their potential symptoms been identified.

The aim of our study was to evaluate the function of the UES across patients with EoE, GERD, and Functional Dysphagia. We also studied the relationship of UES metrics with esophageal symptoms, esophageal body contraction, and EGJ metrics.

## 2. Materials and Methods

### 2.1. Study Sample

Consecutive adult patients with EoE, GERD, or Functional Dysphagia who were recently diagnosed at our outpatient clinic were prospectively enrolled in this pilot study from January 2018 to December 2021. All patients underwent an HRM study.

The diagnosis of EoE was defined, according to recent guidelines, by the following criteria: (1) symptoms of esophageal dysfunction; (2) the presence of ≥15 eosinophils/high-power field (HPF) (or >60 eosinophils/mm^2^) on esophageal biopsy obtained via esophagogastroduodenoscopy (EGD); and (3) exclusion of other potential causes of esophageal eosinophilia [11]. At the baseline, using EGD, the Eosinophilic Esophagitis Endoscopic Reference Score (EREFS) was computed, and six esophageal biopsies were collected from the proximal, middle, and distal esophagus and were histologically evaluated [12,13]. In addition, patients were recently diagnosed and had not started any therapy, such as dietary changes, proton pump inhibitors (PPIs), or topical corticosteroids.

Patients with GERD were diagnosed according to Lyon’s criteria with a positive multichannel intraluminal impedance-pH (MII-pH) monitoring or through EGD findings of grade C or D esophagitis according to the Los Angeles classification [14]. GERD patients were recently diagnosed and off PPI therapy.

**Figure 1:**
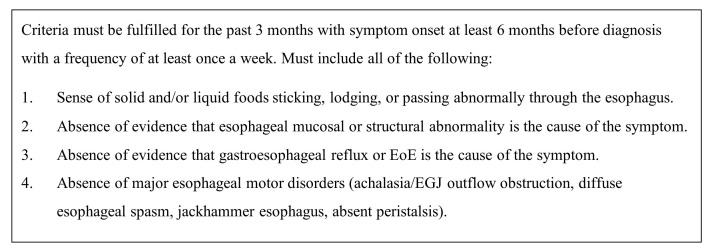
Diagnostic criteria for Functional Dysphagia according to Rome IV criteria.

Inclusion criteria for patients diagnosed with Functional Dysphagia, a disorder of brain–gut interactions (DGBIs) involving the esophagus, were based on Rome IV criteria (Figure 1) [15].

Patients in whom the UES was not well visualized using HRM were excluded from the study.

### 2.2. High-Resolution Manometry and Protocol

Manometry data with impedance was collected using a 2.4 mm diameter solid-state catheter with 36 circumferential pressure sensors spaced 1 cm apart (Unisensor, Laborie, Portsmouth, NH). The catheter was calibrated before use according to the manufacturer’s specifications.

The examination was performed by 3 trained gastroenterologists, L.R., A.S., and P.I., who have at least 5 years of experience. After an overnight fast, the HRM with an impedance catheter was introduced trans-nasally with topical anesthesia. Patients were studied in the semi-recumbent position. Sensors were positioned to record from the hypopharynx, the entire esophagus including the UES, the lower esophageal sphincter (LES), and the stomach. The manometric protocol was performed according to the Chicago Classification 3.0 criteria, with ten 5 mL wet swallows in the supine position [16].

### 2.3. High-Resolution Manometry Data Analysis

**Figure 2:**
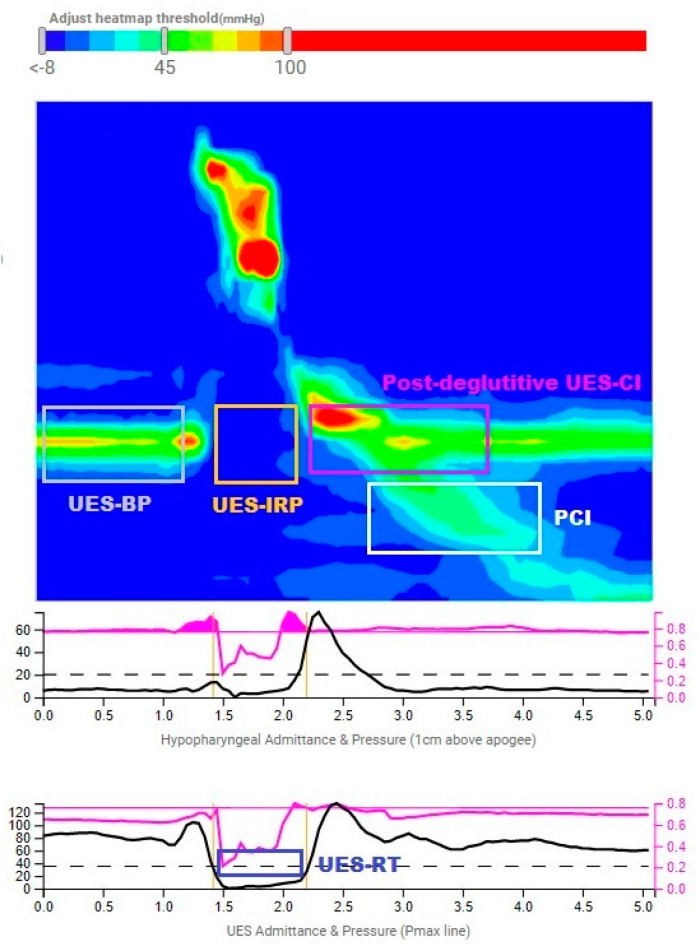
UES metrics on the Swallow Gateway platform.

HRM data were analyzed using the online analysis platform Swallow Gateway (swallowgateway.com, Flinders University, Adelaide, Australia) [17]. After exporting the study data as an ASCII file and uploading it to the web application, spatiotemporal landmarks were manually selected, and HRM metrics were automatically derived. The analysis details and reliability have been previously described, showing, among its strengths, the presence of reference values for pharyngeal analysis [18]. This platform, therefore, is the only one at present that allows us to perform a reproducible and objective analysis of the UES. Figure 2 shows an iconographic representation of the Swallow Gateway platform with UES metrics.

The UES metrics selected included UES Resting Pressure (UES-RP), UES Basal Pressure (UES-BP), UES Integrated Relaxation Pressure (UES-IRP), UES Relaxation Time (UES-RT), Basal UES Contractile Integral (Basal UES-CI), Post-Deglutitive UES Contractile Integral (Post-Deglutitive UES-CI), and Proximal Contractile Integral (PCI). Table 1 shows the technical definitions of the UES metrics [5,19].

Esophageal analysis was performed separately [16]. The selected esophageal body contraction and EGJ metrics were Distal Contractile Integral (DCI), Distal Latency (DL), LES Basal Pressure (LES-BP), LES Contractile Integral (LES-CI), and LES Integrated Relaxation Pressure (LES-IRP) [16].

### 2.4. Standardized Symptoms Questionnaire

A previously published standardized questionnaire dealing with the frequency (0 = absent, 1 = 2 days/week; 2 = 3–5 days/week; and 3 = 6 or 7 days/week) and the intensity (0 = absent; 1 = not very bothersome, not interfering with daily activities; 2 = bothersome, but not interfering with daily activities, and 3 = interfering with daily activities) of the esophageal symptoms, routinely used in our outpatient clinic, was administered on the day of the HRM [20,21,22,23].

Esophageal symptoms were dysphagia for solids, dysphagia for liquids, regurgitation, heartburn, non-cardiac chest pain, asthma, cough, odynophagia, and globus. For each symptom, a frequency–intensity score from 0 up to a maximum of 6 was obtained.

### 2.5. Statistical Analysis

The normality of the distribution of continuous variables was tested by a one-sample Kolmogorov–Smirnov test. Continuous variables with a normal distribution were presented as a mean (standard deviation [SD]); non-normal variables were reported as a median (interquartile range [IQR]). When appropriate, a χ^2^ test to compare the categorical data and an analysis of variance (ANOVA) to compare normally distributed continuous variables were used. Univariate and multivariate analyses were used to appropriately adjust for age, gender, and Body Mass Index (BMI). Spearman rank correlation tests were used to correlate the UES and esophageal body contraction–EGJ metrics, as well as UES metrics and esophageal symptoms. Significance was expressed at a *p* < 0.05 level. SPSS for Windows (release 15.0; SPSS Inc. Chicago, IL, USA) was used for the statistical analysis.

## 3. Results

### 3.1. Patient Characteristics

We recruited 30 patients with EoE, 18 patients with GERD, and 29 patients with Functional Dysphagia. The demographic characteristics are shown in Table 2. Patients with EoE were significantly younger in age than patients with GERD and Functional Dysphagia (*p* = 0.005). The male gender was significantly more prevalent in patients with EoE (*p* < 0.001). BMI was significantly higher in patients with GERD than in patients with EoE and Functional Dysphagia (*p* = 0.001).

### 3.2. UES Metrics

**Figure 3:**
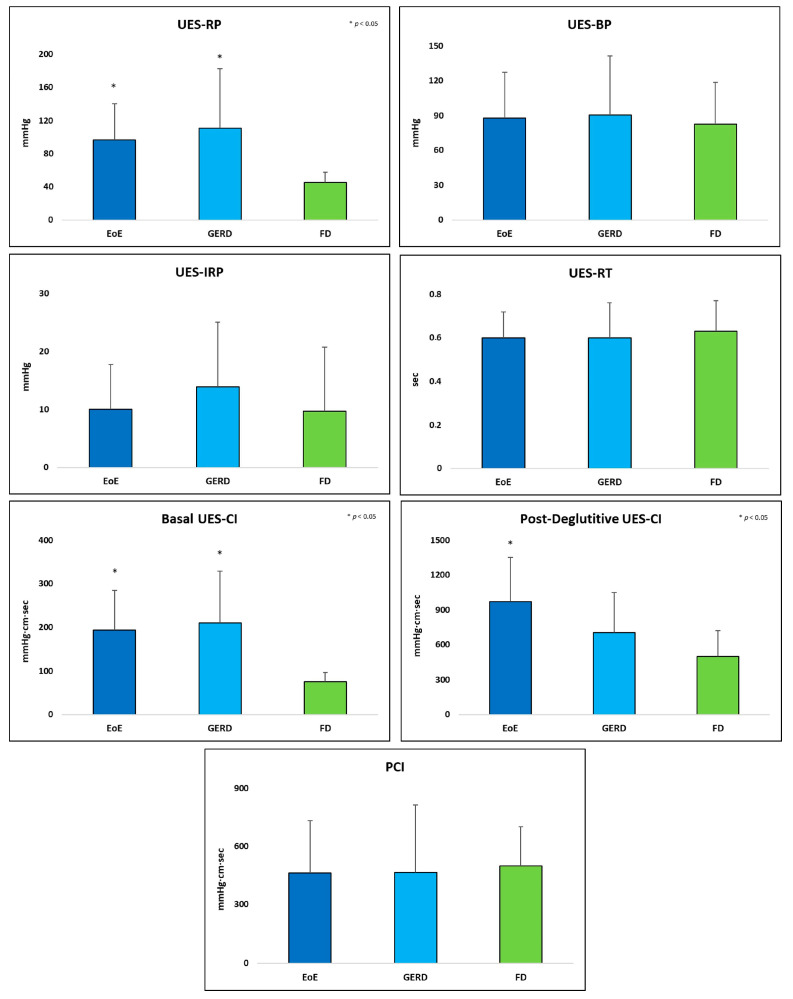
Mean and standard deviation of UES metrics across EoE, GERD, and Functional Dysphagia.

Figure 3 shows the mean and standard deviations of the UES metrics in the three subgroups.

ANOVA analysis adjusted for age, gender, and BMI showed significantly higher values of Post-Deglutitive UES-CI in EoE patients compared with Functional Dysphagia patients (*p* = 0.001). Basal UES-CI and UES-RP showed significantly higher values in EoE patients (*p* = 0.002 and *p* = 0.038) and GERD patients (*p* < 0.001 and *p* = 0.001) compared with Functional Dysphagia patients. The UES-BP, UES-RT, UES-IRP, and PCI did not differ among the three subgroups.

Multivariate regression analysis, with Post-Deglutitive UES-CI, UES-RP, and Basal UES-CI as dependent variables, and age, gender, and BMI as covariates, demonstrated that Post-Deglutitive UES-CI was significantly higher in EoE patients compared to Functional Dysphagia patients (B = 461.431 (229.6–693.3), *p* < 0.001). Basal UES-CI and UES-RP were significantly higher in EoE (B = 108.013 (48.1–167.9), *p* = 0.001 and B = 42.188 (9.4–75.0), *p* = 0.013 respectively) and GERD patients (B = 151.179 (88.3–214.0), *p* < 0.001 and B = 67.588 (33.2–101.9), *p* < 0.001 respectively) compared to Functional Dysphagia patients.

The other UES metrics that were analyzed showed no statistically significant differences across the EoE, GERD, and Functional Dysphagia groups.

Four EoE patients had a GERD diagnosis according to the Lyon Consensus. A further statistical analysis excluding these patients did not show any significant changes (Supplementary Table S2).

### 3.3. Esophageal Body Contraction and EGJ Metrics

Supplementary Table S1 shows the results of the esophageal analysis in the three groups using a univariate analysis adjusted for age, gender, and BMI. LES-BP was significantly higher in EoE patients than in GERD patients (*p* = 0.024).

A bivariate correlation analysis showed a significant correlation between LES-CI and the Post-Deglutitive UES-CI, Basal UES-CI, UES-RP (Rs = 0.468, *p* ≤ 0.001, Rs = 0.266, *p* = 0.027, and Rs = 0.285, *p* = 0.017, respectively), and between LES-BP and Post-Deglutitive UES-CI (Rs = 0.281, *p* = 0.019), independent of diagnosis.

### 3.4. Esophageal and Extraesophageal Symptoms

**Figure 4:**
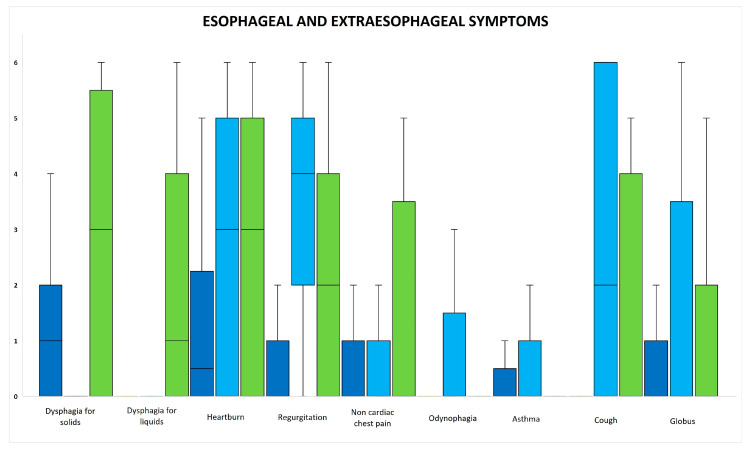
Box plot of esophageal and extraesophageal symptoms of EoE (blue), GERD (light blue) and Functional Dysphagia (green) patients.

Figure 4 shows a box plot of the esophageal and extraesophageal symptoms. In EoE patients, there was a high incidence of dysphagia for solids, regurgitation, and globus. In GERD patients, there was a high incidence of heartburn, regurgitation, odynophagia, cough, and globus. In Functional Dysphagia patients, there was a high incidence of dysphagia for solids, dysphagia for liquids, heartburn, regurgitation, non-cardiac chest pain, cough, and globus.

A cumulative symptom score of all symptoms was computed. There was a significantly lower cumulative score in patients with EoE compared to those with GERD and Functional Dysphagia.

Bivariate correlation analysis between the UES, EGJ metrics, and esophageal and extraesophageal symptoms showed no statistically significant correlation across the EoE, GERD, and Functional Dysphagia groups.

## 4. Discussion

To our knowledge, the present study is the first to analyze UES metrics across EoE, GERD, and Functional Dysphagia patients.

Our main results were that Post-Deglutitive CI was significantly higher in patients with EoE than in patients with Functional Dysphagia and Basal UES-CI and UES-RP were significantly higher in patients with EoE and GERD than in patients with Functional Dysphagia. Moreover, these three UES metrics had a significant correlation with LES-CI, while Post-Deglutitive CI correlated with LES-BP. The cumulative esophageal symptom score was significantly lower in patients with EoE than in GERD and Functional Dysphagia patients.

The pathophysiology and the following clinical implications of conditions such as EoE and GERD on UES function are still unclear. Innovations in the field of HRM have made the study of the UES much easier, although, to date, there is a lack of international guidelines standardizing UES metrics and how to measure them.

Currently, few studies have analyzed the UES in patients with GERD, and they have demonstrated contrasting results. A recent study found a shorter and hypotonic UES, especially in patients with extraesophageal symptoms [7,24]. Other studies found an increased UES tone, and a mechanism of aspiration protection has been hypothesized. [25,26]. Our results, which show higher values of Basal UES-CI and UES-RP, might confirm the hypothesis of a protective mechanism of reflux-related aspiration.

Moreover, a significant but mild correlation between UES metrics and LES-CI was revealed in this study. It has been already demonstrated that LES-CI might differentiate the severity of distal esophageal acid exposure better than inspiratory EGJ pressure, expiratory EGJ pressure, and the degree of separation between the LES and CD [27]. Thus, it is possible to speculate that a positive correlation between UES metrics and LES-CI can be explained as further strengthening the barrier against reflux. However, this hypothesis should be confirmed by future studies with larger sample sizes and healthy controls.

Currently, no study has analyzed UES metrics in patients with EoE. In our study, higher values of Basal UES-CI, UES-RP, and Post-Deglutitive UES-CI were found in patients with EoE. The results obtained for UES metrics in patients with EoE can be explained by the changes in the mechanical propriety of the esophagus that occur in this disease. Tissue remodeling with basal zone hyperplasia [28], smooth muscle hyperplasia and hypertrophy [29], mucosal vascular proliferation [30], and fibrostenotic changes [31] were documented. These pathophysiologic changes appear to underlie the alterations found at the functional luminal imaging probe (endo-FLIP), with less esophageal distensibility compared with controls [32].

In addition to anatomical changes, uncoordinated esophageal peristalsis has also been hypothesized. Abundant full-thickness eosinophilic infiltration that affects the muscularis mucosa and muscularis propria has been demonstrated in esophagectomy specimens from patients with EoE [33]. EUS studies evaluating real-time muscle contraction during peristalsis have found alterations in the longitudinal muscles of the esophagus in patients with EoE [34]. The eosinophils in patients with EoE could also have a direct impact on the dynamic function of the esophageal muscle. Both eosinophils and mast cells release mediators that affect muscle function, resulting in increased contraction (e.g., leukotriene D4, prostaglandin F2 alpha) or relaxation (e.g., IL-6, IL-13) [35,36,37]. Degranulation of eosinophils also results in the production of neurotoxic mediators, including eosinophil cationic protein and eosinophil-derived neurotoxin [38]. Given the established deep infiltration of eosinophils into the esophagus of patients with EoE, including into smooth muscle and the myenteric plexus, it is hypothesized that these mediators cause dysfunction and/or destruction of neurons that may contribute to dysmotility [39].

Thus, our hypothesis is that the combination of these anatomical and motor alterations might slow down esophageal peristalsis during swallowing, resulting in esophageal dilatation and hyperactivation of reflex responses that result in greater upstream contraction and a higher Post-Deglutitive UES-CI value. Similarly, the increased wall stiffness observed in patients with EoE also results in increased pressures observed in the resting period with higher Basal UES-CI and UES-RP values.

To our knowledge, there are no studies of the UES function in patients with disorders of DGBIs involving the esophagus. The results of the UES metrics were all within the normal limits imposed by the Swallow Gateway platform.

No correlation between UES, EGJ metrics, esophageal and extraesophageal symptoms, and the three diagnostic subgroups was found. However, the cumulative symptom score was significantly lower in patients with EoE than in GERD and Functional Dysphagia patients, confirming an increasing spectrum of symptoms from true GERD to increased esophageal perception of Functional Dysphagia patients. Only one study analyzed some concomitant esophageal symptoms in Functional Dysphagia patients, confirming a high frequency of regurgitation and noncardiac chest pain [40].

Our study has several limitations. First, although this was a pilot study and EoE is a rare disease with a very long diagnostic delay, a low number of patients was selected [41]. Second, the significantly lower age and the higher male prevalence of EoE patients compared with GERD and Functional Dysphagia patients could explain the increased tone and CI of UES; this is probably due to the presence of compensatory mechanisms that weaken with advancing age and the presence of greater reflex responses in males. However, in the multivariate analysis, these results were confirmed even when adjusted for age and sex. Third, this study lacked healthy volunteers as a control group; however, the UES, esophageal, and EGJ metrics of the patients were within the normal values of the Swallow Gateway platform, which is an internationally adopted platform that is supported by growing scientific evidence [42,43]. Fourth, there is a lack of standardized guidance for the UES analysis regarding the amount of fluid to be administered and the patient’s position during swallowing. However, this is a pilot study that started in 2018, and the standard protocol was performed according to the Chicago classification 3.0. We are developing new studies that will consider both positions as suggested by the Chicago classification 4.0. Fifth, we have not evaluated the pharyngeal metrics or the impedance of the UES in these groups of patients; however, we hope to carry out these measurements in a future study.

## 5. Conclusions

In conclusion, our pilot study showed differences in UES metrics in EoE, GERD, and Functional Dysphagia patients. Future studies are needed to investigate whether these differences in UES metrics in EoE, GERD, and FD patients have any significant clinical implications.

## Figures and Tables

**Table 1:** Descriptive terminology, acronyms, and technical definitions for HRM outcomes.

Metric	Acronym (Units)	Definition
UES Resting Pressure	UES-RP (mmHg)	Mean of UES axial maximum pressures from the proximal limit of the UES to the distal point of the sphincter for three consecutive respiratory cycles [5]
UES Basal Pressure	UES-BP (mmHg)	Mean of UES axial maximum pressures preceding UES relaxation [19]
UES Integrated Relaxation Pressure	UES-IRP (mmHg)	The extent of UES relaxation defined as the median of the lowest pressures in a nonconsecutive window of 0.25 s [19]
UES Relaxation Time	UES-RT (s)	Duration of UES relaxation defined as the interval when pressure is <50% of baseline or <35 mmHg (the lowest) [19]
Basal UES Contractile Integral	Basal UES-CI (mmHg∙cm∙s)	The integral of pressures from the proximal limit of the UES to the distal point of the sphincter for three consecutive respiratory cycles [5]
Post-Deglutitive UES Contractile Integral	Post-Deglutitive UES-CI (mmHg∙cm∙s)	The integral of pressures of the UES post-swallow, indicating UES contractile vigor [19]
Proximal Contractile Integral	PCI (mmHg∙cm∙s)	The integral of pressures > 20 mmHg within the proximal esophagus region, indicating contractile vigor of the proximal esophagus [19]

**Table 2:** Demographic characteristics.

	EoE	GERD	FD	*p*-Value
Number (%total)	30 (39.0%)	18 (23.4%)	29 (37.7%)	-
Age (years, M ± SD)	39.1 ± 13.2	52.1 ± 15.2	51.7 ± 14.6	0.005
Gender (%M)	27 (90%)	9 (50.0%)	12 (41.4%)	<0.001
BMI (Kg/m^2^, M ± SD)	24.7 ± 3.2	29.0 ± 5.0	24.7 ± 3.9	0.001

## Data Availability

Not applicable.

## Supplementary Materials

The following supporting information can be downloaded at: https://www.mdpi.com/article/10.3390/jcm12175548/s1, Table S1: Mean and standard deviation of esophageal HRM outcomes and alterations in esophageal motility. Table S2: ANOVA analysis and post hoc test excluding patients with EoE who also had a diagnosis of GERD.
