# Supplementary material for: Upper Esophageal Sphincter Metrics across Eosinophilic Esophagitis, Gastroesophageal Reflux Disease and Functional Dysphagia: A Pilot Study
Source: J Clin Med. 2023 Aug 25;12(17):5548. doi: 10.3390/jcm12175548 (PMC10488079; doi:10.3390/jcm12175548)
Supplement: Supplementary file 1 [file jcm-12-05548-s001.zip › jcm-2522284-supplementary.pdf]

Supplementary Table S1. Mean and standard deviation of esophageal HRM outcomes and alterations in esophageal motility.

	EoE	GERD	FD	p-value
**DCI (mmHg·cm·sec)**	1476.9 ± 887.7	1257.7 ± 1003.5	2223.7 ± 1344.0	0.244
**DL (sec)**	6.9 ± 1.0	7.0 ± 0.9	6.8 ± 0.9	0.610
**LES-BP (mmHg)**	71.2 ± 41.3	31.7 ± 19.2	39.3 ± 21.3	**0.019**
**LES-IRP (mmHg)**	11.9 ± 10.1	13.9 ± 12.5	10.9 ± 8.4	0.751
**LES-CI (mmHg·cm·sec)**	163.1 ± 103.9	105.9 ± 123.1	86.1 ± 85.4	0.196
**LES-IRP pathological, N (%)**	5 (16.7)	n/a	n/a	
**≥50% ineffective swallows (Chicago 3.0), N (%)**	6 (20.0)	n/a	n/a	

Supplementary Table S2. ANOVA analysis and test post hoc excluding patients with EoE who also had a diagnosis of GERD.

	EoE	GERD	FD	p-value
**UES-RP (M ± SD)**	99.0 ± 46.1 [a]	110.7 ± 71.7 [b]	43.8 ± 13.7 [a,b]	**< 0.001**
**UES-BP (M ± SD)**	90.9 ± 39.7	90.5 ± 50.9	79.6 ± 37.7	0.605
**UES-IRP (M ± SD)**	9.9 ± 8.1	13.9 ± 11.1	9.6 ± 11.1	0.363
**UES-RT (M ± SD)**	0.6 ± 0.1	0.6 ± 0.2	0.6 ± 0.1	0.720
**Basal UES-CI (M ± SD)**	196.3 ± 96.7 [a]	210.1 ± 118.4 [b]	72.4 ± 23.7 [a,b]	**< 0.001**
**Post-deglutitive UES-CI (M ± SD)**	940.2 ± 377.3 [a]	701.6 ± 349.5	480.0 ± 232.7 [a]	**< 0.001**
**PCI (M ± SD)**	459.1 ± 257.1	464.3 ± 348.5	482.5 ± 212.7	0.954

[a] $p<0.05$ EoE vs FD, [b] $p<0.05$ GERD vs FD
